# Older Adult Fatality Reviews: A Powerful Tool to Improve Policies, Protections, and Services

**DOI:** 10.1093/geroni/igaa057.2458

**Published:** 2020-12-16

**Authors:** Janet Wilson, Kimethria Jackson

**Affiliations:** 1 University of Oklahoma Health Sciences Center, Reynolds Center for Geriatric Nursing Excellence, Oklahoma City, Oklahoma, United States; 2 University of Oklahoma Health Sciences Center, Oklahoma City, Oklahoma, United States

## Abstract

Older adult maltreatment is a serious problem that can hasten mortality and cause fatality. Child and Domestic Violence Fatality Review teams have made a major impact to improve protections, services, and system responses for victims. There are over 1300 child fatality review teams in 50 US states and 200 domestic violence fatality teams in 45 states. Older adult fatality reviews, in contrast, have not proliferated across the country, missing opportunities to bring interdisciplinary expertise together to resolve policy, protection, and services needed to prevent older adult premature deaths due to violence and abuse. Method: A mini mock review of a fatality of an older adult woman will demonstrate the possibilities of beginning Older Adult Fatality Review Board as recommended by the American Bar Association. Conclusion: Interdisciplinary older adult fatality review teams have been an underutilized tool to make changes in older adult mistreatment protection services and systems.

